# Reciprocal Interactions of Human Monocytes and Cancer Cells in Co-Cultures In Vitro

**DOI:** 10.3390/cimb46070408

**Published:** 2024-07-02

**Authors:** Roman Paduch, Maria Klatka, Paulina Pieniądz, Iwona Wertel, Anna Pawłowska, Janusz Klatka

**Affiliations:** 1 Department of Virology and Immunology, Institute of Biological Sciences, Faculty of Biology and Biotechnology, Maria Curie-Skłodowska University, Akademicka 19, 20-033 Lublin, Poland; paulina.pieniadz@mail.umcs.pl; 2 Department of General and Paediatric Ophthalmology, Medical University of Lublin, Chmielna 1, 20-079 Lublin, Poland; 3 Department of Paediatric Endocrinology and Diabetology, Medical University, Gębali 1, 20-093 Lublin, Poland; maria.klatka@umlub.pl; 4 Independent Laboratory of Cancer Diagnostics and Immunology, Medical University of Lublin, Chodźki 1, 20-093 Lublin, Poland; iwona.wertel@umlub.pl (I.W.); anna.pawlowska@umlub.pl (A.P.); 5 Department of Otolaryngology and Laryngological Oncology, Medical University of Lublin, Jaczewskiego 8, 20-954 Lublin, Poland; janusz.klatka@umlub.pl

**Keywords:** cancer, monocytes/macrophages, co-culture, cellular interactions

## Abstract

The tumor microenvironment (TME) includes immune and stromal cells and noncellular extracellular matrix (ECM) components. Tumor-associated macrophages (TAMs) are the most important immune cells in TME and are crucial for carcinomas’ progression. The purpose was to analyze direct and indirect interactions in co-culture of tumor cells with monocytes/macrophages and, additionally, to indicate which interactions are more important for cancer development. Cytokines, reactive oxygen species, nitric oxide level, tumor cell cycle and changes in tumor cell morphology after human tumor cells (Hep-2 and RK33 cell lines) with human monocyte/macrophage (THP-1 cell line) interactions were tested. Morphology and cytoskeleton organization of tumor cells did not change after co-culture with macrophages. In co-culture of tumor cells with human monocyte, changes in the percentage of tumor cells in cell cycle phases was observed. No significant changes in reactive oxygen species (ROS) were found in the co-culture as compared to the tumor cell mono-culture. Monocytes produced about three times higher ROS than tumor cells. In co-cultures, a lower nitric oxide (NO_x_) level was found as compared to the sum of the production by both mono-cultures. Co-culture conditions limited the production of cytokines (IL-4, IL-10 and IL-13) as compared to the sum of their level in mono-cultures. In conclusion, macrophages influence tumor cell growth and functions. Mutual (direct and paracrine) interactions between tumor cells and macrophages changed cytokine production and tumor cell cycle profile. The data obtained may allow us to initially indicate which kind of interactions may have a greater impact on cancer development processes.

## 1. Introduction

Most cancers belong to squamous cell carcinomas. Tumor-infiltrating cells and the development of the local microenvironment play an important role in the development and dissemination of head and neck cancer cells. In addition, direct and paracrine interactions of tumor cells with infiltrating immunologically competent cells are also important. Therefore, focusing on intercellular relationships in the local tumor microenvironment (TME) is valid in determining the course of disease and ways to limit it [1]. The tumor microenvironment consists of cancerous cells, endothelial cells included in the composition of blood or lymphatic vessels, immune cells, fibroblasts and the extracellular matrix (ECM). In the group of immune cells composing and infiltrating tumor mass, subpopulations of lymphocytes T, lymphocytes B, NK cells, dendritic cells, myeloid-derived suppressor cells (MDSC) and macrophages should be distinguished. All of them play an important role in the growth regulation and dissemination of tumor cells [1,2]. However, macrophage-infiltrating cancers and tumor-associated macrophages (TAMs) are the most important immune cells shaping the tumor microenvironment. They account up to 50% of the total inflammatory cell population or half of the total weight of the pathological mass [3,4]. TAMs are determined by their two polarization states: classically activated M1 and alternatively activated M2 phenotypes [5,6]. M1 forms promote tumor immunity, while M2 forms tumor inflammation [2]. Polarization to the appropriate form depends mainly on soluble mediators which are present in the local microenvironment. Macrophages localized near blood vessels polarize to M1, but polarize also away from vessels under hypoxic conditions and transform into subtype M2 [7]. It is therefore considered that TAMs in tumor masses are very heterogenous populations and express functionality and typical markers of both macrophage subtypes [6]. These cells interact with cellular and noncellular elements composing tumor, leading to the promotion of immunosuppressed and inflammatory conditions. As a consequence, it makes it easier for cancer cells to infiltrate and metastasize [3,8]. The speed of these processes depends on both the quality of direct and indirect interactions through soluble mediators between TAMs and cancer cells. Cellular dialogue related to the interaction of specific surface receptors or the cell cytoskeleton as well as cytokines (pro- and anti-inflammatory), growth factors, reactive oxygen species (ROS) or nitric oxide (NO_x_) released to pericellular space may determine the aggressiveness of a cancer and thus be essential in tumor progression [2,9]. Cytokines such as IL-4, which stimulate macrophage/monocyte activity or is involved in inflammatory state activation, and IL-10 and IL-13, exhibiting immunoregulatory and anti-inflammatory activity, are considered to be important in reciprocal interactions between tumor cells and macrophages in co-culture systems [10]. Fluctuating cytokine levels also affect the release of NO_x_ and ROS in co-cultures and thus regulate further tumor development. As a consequence, changes in parameters of soluble mediators during intercellular interactions (cancer macrophages) may have a prominent role in tumor progression, promotion of EMT and finally metastasis [11]. Knowledge of these interactions can be the basis for identifying new prognostic parameters, molecular therapeutic targets or, generally, the preparation of new effective therapeutic strategies focused on limiting mutual stimulation by immune and cancer cells. The aim of the presented paper was to determine the differences in the level of released factors (reactive oxygen species, cytokines and nitric oxide) and cellular changes (differences in the cell cycle, cytoskeletal structure and general morphology of cancer cells) in direct and indirect co-culture of human cancer cells with macrophages. The aim was also to indicate which interactions are more important from the point of view of cancer development.

## 2. Materials and Methods

### 2.1. Cell Line

An RK33 cell line was established, using the liquid overlay method, in the Department of Virology and Immunology, UMCS, Lublin, Poland, from a tumor fragment at stage T3 taken from a 50-year-old woman after laryngectomy [12]. Culture was carried out at 37 °C in a humidified atmosphere with 5% CO_2_ in RPMI 1640 medium supplemented with 10% fetal bovine serum (FBS) (Gibco, Paisley, UK) and antibiotics (100 U/mL penicillin, 100 μg/mL streptomycin) in a humidified atmosphere with 5% CO_2_.

Cells of the human carcinoma line (Hep-2: ECACC Cat. No. 86030501) were grown in 25 cm^2^ culture flasks (Nunc., Roskilde, Denmark) in RPMI 1640 medium supplemented with 10% fetal bovine serum (FBS) (Gibco, Paisley, UK) and antibiotics (100 U/mL penicillin, 100 μg/mL streptomycin) at 37 °C in a humidified atmosphere with 5% CO_2_. The Hep-2 cell line, that was originally derived as carcinoma of the larynx, contains HeLa marker chromosomes, and the tested cell line could then be HeLa-contaminated.

The suspension human myelomonocytic cell line (THP-1: ECACC Cat. No. 88081201) was carried out in RPMI 1640 medium supplemented with 10% fetal bovine serum (FBS) at 37 °C in a humidified atmosphere with 5% CO_2_.

### 2.2. Experimental Design

In the presented study, two human cancer cell lines (Hep-2 and RK33) and cells from the human monocytic leukemia cell line THP-1 were used. Mono-cultures and co-cultures of cells were prepared. Experiments were performed in two models of cell interactions: direct contact of tumor and monocyte cells and indirect contact in inserts, where both kind of cells were separated with a polycarbonate membrane (0.4 μm). A cell density of 1 × 10^5^ cells/mL was applied in all experiments. In the co-cultures model of direct interactions, a mixture of tumor cells and monocytes in a ratio of 1:0.3 was used. Thus, THP-1 cells accounted for 30% of the tumor cell density. In the indirect setup, monocytes were placed in inserts, and cancer cells were cultured at the bottom of a 24-well plate. Inserts seeded with monocytes (THP-1) (300 μL) were placed inside the wells of 24-well plates with tumor cells on the bottom and filled with 700 μL of medium. After 24 h of incubation at 37 °C, 5% CO_2_ in a humidified incubator, the medium and cells were collected for further analyses.

### 2.3. Lactate Dehydrogenase (LDH) Assay

Lactate dehydrogenase (LDH) belongs to oxidoreductase enzymes catalyzing the conversion of pyruvate and lactate. This test is based on the reduction of NAD by LDH. The reduced form of NAD is used in the stoichiometric conversion of a tetrazolium dye. The colored compound, which was formed after specific reactions, was measured spectrophotometrically at 570 nm.

A whole assay procedure was performed according to the manufacturer’s instruction. Briefly, Lactate Dehydrogenase Assay Mixture (LDH Assay Substrate Solution, LDH Assay Dye Solution and LDH Assay Cofactor) was prepared fresh just directly before use. To the culture medium sample (50 μL), Lactate Dehydrogenase Assay Mixture (100 μL) was added. Next, protection from light incubation was performed for 30 min at room temperature. The reaction was terminated using 15 μL 1 N HCl, added to each well of the 96-well plate. The optical density was measured at the primary wavelength of 490 nm using a microplate reader (BioTek Instruments, Winooski, VT, USA).

### 2.4. Nitric Oxide (NO) Measurement

For analysis, the spectrophotometric Griess method was applied. Nitrate, which is a stable end product of NO, was tested in the culture supernatants. After 24 h of incubation of both kind of cells, the culture medium was collected for analysis. Briefly, 100 μL of the supernatant was plated in 96-well flat-bottomed plates in triplicate and incubated with 100 μL of Griess reagent (1% sulphanilamide/0.1% N-(1-naphthyl)ethylenediamine dihydrochloride) (Sigma, St. Louis, MO, USA) in 3% H_3_PO_4_ (POCH Gliwice, Poland) at room temperature for 10 min. The optical density was measured at 550 nm using a microplate reader (BioTek Instruments, Winooski, VT, USA). A standard curve was prepared using 0.5–25 μM sodium nitrite (NaNO_2_) for calibration and quantitative estimation of NO concentration in the tested samples.

### 2.5. May-Grünwald–Giemsa (MGG) Staining

The MGG staining enabled us to visualize the morphology of cells and their potential changes.

The tumor cells at a density of 1 × 10^5^ cells/mL were cultured in Petri dishes (35 mm). In co-culture, the amount of macrophages accounted for 30% of the amount of tumor cells. After 24 h of incubation, the medium was discarded, and cells were fixed with the May-Grünwald dye containing methanol for 5 min. Next, the dye was diluted in an equal volume of water and incubated for 2 min. Thereafter, the dye was removed, and Giemsa stain, previously diluted (1 vol. Giemsa: 19 vol. water), was added for 20 min. The preparations were profusely rinsed three times with distilled water and dried in room temperature. The observation of cells was performed under a light microscope (Olympus BX51, Tokyo, Japan).

### 2.6. ELISA Assay

The concentration of human IL-4, IL-10 and IL-13 were measured immunoenzymatically (ELISA) using commercially available kits according to the manufacturer’s instruction (Biorbyt, Cambridge, Cambridgeshire, UK). Briefly, 100 μL of samples were added to appropriate plate wells. After 2 h of incubation, and a series of washing, enzyme-conjugated secondary antibodies (100 μL) were added to the wells and incubation was continued for 1 h. After washing, 100 μL of the enzyme substrate was added to the wells for detection. After 30 min of incubation, the color reaction was stopped by adding 2M H_2_SO_4_ to each well. The optical density of the colored product was determined using a microplate reader (BioTek Instruments, Winooski, VT, USA) at 450 nm. The concentrations of the cytokines in the samples were calculated on the basis of a parallelly prepared standard curve. The detection limit was 3.5 pg/mL (IL-4 and IL-10) and 7.1 pg/mL (IL-13).

### 2.7. Labelling of Cytoskeletal F-Actin Filaments

Cell cytoskeleton visualization was performed using the fluorescent Rhodamine–phalloidin staining method. Briefly, the cells (mono-cultures and direct co-cultures) were incubated in 4-well Lab-Tek chamber slides in RPMI 1640 culture medium supplemented with 2% FCS. After 24 h incubation, the cells were fixed with 10% paraformaldehyde for 20 min, rinsed profusely 3 times with PBS and exposed to 0.2% Triton X-100 for 5 min. After rinsing, 1 mg/mL TRITC (tetramethyl Rhodamine isothiocyanate)–phalloidin (Sigma, St. Louis, MO, USA) in PBS was added to the preparations, and incubation was performed for 30 min in the dark at 37 °C/5% CO_2_. The cells were rinsed 3 times with PBS and assessed under a fluorescence microscope (Olympus BX-51, Tokyo, Japan). The analysis of the fluorescent images was performed using the imaging software program AnalySIS 2.0.

### 2.8. Assessment of ROS Using 2′,7′-Dichlorodihydrofluorescein Diacetate (H_2_DCF/DA)

ROS production in co-culture of human tumor cells with human monocytic leukemia cells was measured using the fluorogenic probe H_2_DCF/DA. H_2_DCF/DA is a fluorescent dye that easily crosses the cell membrane. Within cells, it is hydrolyzed by cellular esterases to the form of 2′,7′-dichlorodihydrofluorescein (H_2_DCF). After de-acylation, the probe is susceptible to oxidation and generates a fluorescent product, 2′,7′-dichlorofluorescein DCF. An accumulation of DCF indicates the production of redox-active compounds which can be measured under a confocal scanning microscope [13].

After 24 h of co-culture, the cells were washed with PBS containing Ca^2+^ and Mg^2+^ and were treated with H_2_DCF at final concentration (4 μM). The cells were then incubated for 1 h at 37 °C, 5% CO_2_. Thereafter, the cells were washed with PBS and analyzed under a fluorescence microscope (Olympus BX51, Tokyo, Japan) at an excitation wavelength of 488 nm. The emitted fluorescent light was passed through a 530 nm filter. Imaging analysis was carried out with the imaging software system AnalySIS 2.0.

### 2.9. Cytometric Analysis of the Cell Cycle

The distribution of cells in the cell cycle phases was analyzed using flow cytometry (FCM). The direct co-cultures were prepared in 24-well plates, and the indirect co-cultures were prepared in inserts. Then, incubation was performed for 24 h. As the control, the mono-cultures of both tumor and monocyte cells were used. After incubation, floating and adherent cells were harvested, centrifuged (720× *g*/5 min) rinsed in PBS without Ca^2+^ and Mg^2+^ ions, centrifuged again, and fixed in 70% ethanol. The samples were stored for 1 week at −20 °C. Subsequently, the samples were stained with propidium iodide (PI) (PI/RNase Staining Buffer, BD Pharmingen^TM^, BD Biosciences, San Jose, CA, USA). The PI fluorescence intensity was measured using FACS Calibur (BD Biosciences Pharmingen, San Diego, CA, USA), and the data were analyzed using Cell Quest Pro Version 6.0. for the Macintosh operating system (BD Biosciences Pharmingen, San Diego, CA, USA). The results were calculated as the % of cells in the appropriate cell cycle phases (sub-G1, G0/G1, S and G2) among all the analyzed, gated cells. A total of 10,000 events were measured per sample.

### 2.10. Statistical Analysis

The results are presented as the means ± SD of three independent experiments (n = 3). The data were analyzed using a one-way analysis of variance ANOVA, followed by Dunnett’s multiple comparison post hoc test. Differences were considered significant at *p* ≤ 0.05.

## 3. Results

### 3.1. LDH Release in Co-Cultures

It has been observed that, in both direct and indirect co-cultures of Hep-2 tumor cells with THP-1 monocytes, there was a decrease in the amount of LDH released as compared to the mono-culture of Hep-2 tumor cells. In indirect co-culture of these cells, this decrease was significantly lower (5.44%) (almost eight-fold) than in the case of direct co-culture (Table 1). On the other hand, in both direct and indirect co-cultures of RK33 tumor cells with THP-1 monocytes, there was an increase in the amount of LDH released as compared to the mono-culture of RK33 tumor cells. In the indirect co-culture of these cells, an increase was twice as high (by 6.29%) as compared to the direct co-culture (Table 1).

These results indicate that processes limiting the development of tumor cells may take place in the co-culture system.

### 3.2. Cell Cycle

Co-culture of human tumor cells (Hep-2 or RK33 lines) with human monocytes (THP-1 line) modulated the percentage of tumor cells in cell cycle phase–flow cytometry.

**Figure 1:**
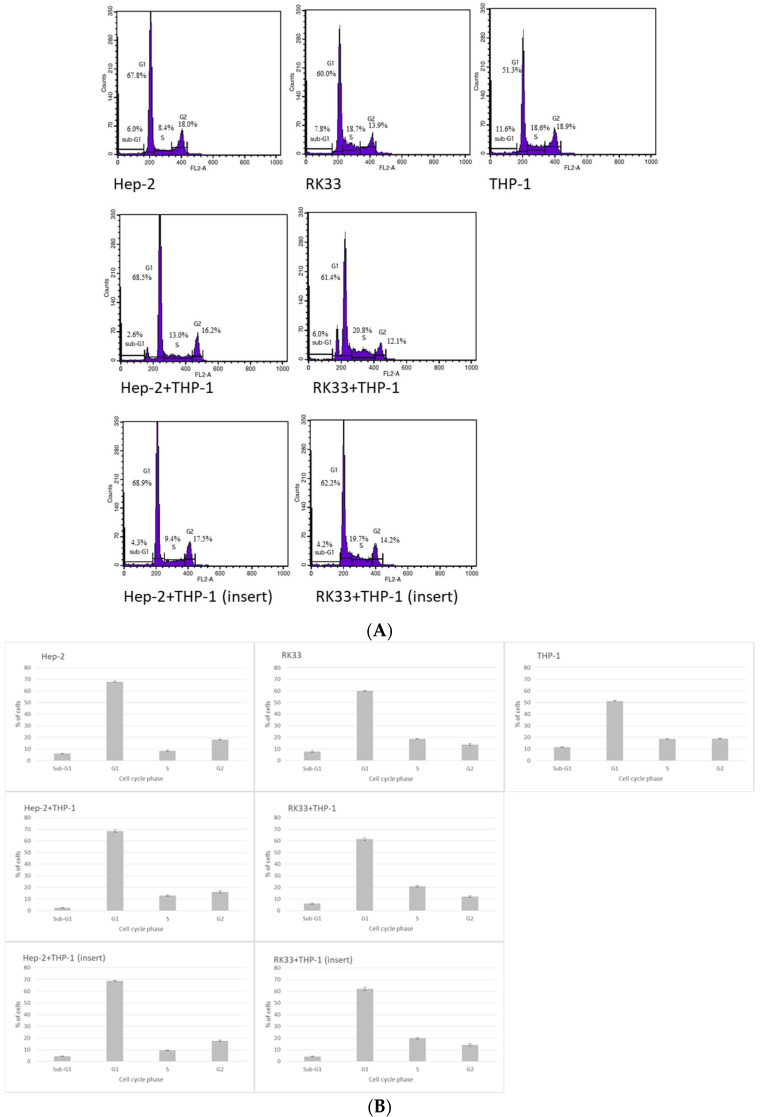
The differences in cell cycle distribution in human tumor Hep-2 and RK33 cells and human monocytic THP-1 cell culture and the co-culture of monocyte and tumor cells analyzed by flow cytometry. The co-cultures were performed in direct tumor/monocyte or indirect (insert) reciprocal interactions. The experiments were conducted for 24 h. The cells were thereafter stained with propidium iodide and analyzed by flow cytometry. Representative DNA histograms for tested cell lines in mono- and co-cultures (**A**) and graphical representations of changes in specific cell phases are shown (**B**). Two peaks that appeared in the G1 phase of the cycle, during direct interactions in co-culture, resulted from different granularity and size of the nuclei of both types of cells.

To assess the influence of direct or indirect interactions of human tumor Hep-2 or RK33 cells with human normal monocyte/macrophage THP-1 cells on the distribution of tumor cell cycle phases after 24 h of co-culture, flow cytometry was used (Figure 1).

Direct co-culture of Hep-2 or RK33 cells with THP-1 monocytes/macrophages significantly decreased the number of tumor cells found in the sub-G1 phase which may represent dying cells, or more specifically, entering apoptosis. In the direct co-culture of THP-1 cells with Hep-2 cells, the number of cell fractions decreased by 56.8%, and with RK33 cells, it decreased by 22.6% as compared to the mono-culture of the corresponding tumor cells. On the other hand, in the case of indirect interactions between both cell types (insert), a reduction in the sub-G1 phase was also observed in the population of tumor cells. However, for Hep-2 cells, the decrease was 28.9%, and for RK33 cells, it was 46.6% as compared to mono-cultures of tumor cells (Table 2).

Both direct and indirect interactions of neoplastic cells with normal monocytes/macrophages did not affect the quantitative changes in the G1 fraction of the interphase cycle as compared to the mono-cultures of transformed cells. The G1 phase is a time that varies for individual cell types, usually lasting about 12 h for mammalian cells. There is intensive synthesis of RNA, structural and regulatory proteins, including cyclins, cell mass and volume increase, and a replication complex is formed. On the histogram of direct interactions in co-culture, two peaks appear in this phase of the cycle, which result from different granularity and size of the nuclei of both types of cells. On the other hand, in indirect interactions, the influence of factors secreted by THP-1 cells on the G1 phase of cancer cells was of importance.

Direct epithelial–macrophagic interactions increased the number of cells entering the S phase of the cell cycle. For the co-culture with Hep-2 cells, the increase was 54.2%, and for the co-culture with RK33 cells, it was 11.6% as compared to the corresponding tumor cell mono-cultures. In the case of indirect co-culture, there was also an increase in the number of neoplastic cells entering the S phase of the cycle, but the increase was lower than that observed in the case of direct interactions. These increases were 12.4% and 5.7%, respectively, in relation to the respective mono-cultures of tumor cells (Table 2).

There was also a decrease in the number of cells in the G2 phase in the case of direct co-culture (Hep-2/THP-1 by 9.9%, RK33/THP-1 by 13.5%) compared to the corresponding tumor cell mono-cultures. In the case of indirect co-culture interactions, the G2 fraction of neoplastic cells did not change significantly as compared to the mono-cultures of transformed cells (Table 2).

### 3.3. IL-4, IL-10 and IL-13 Levels in Co-Cultures

**Figure 2:**
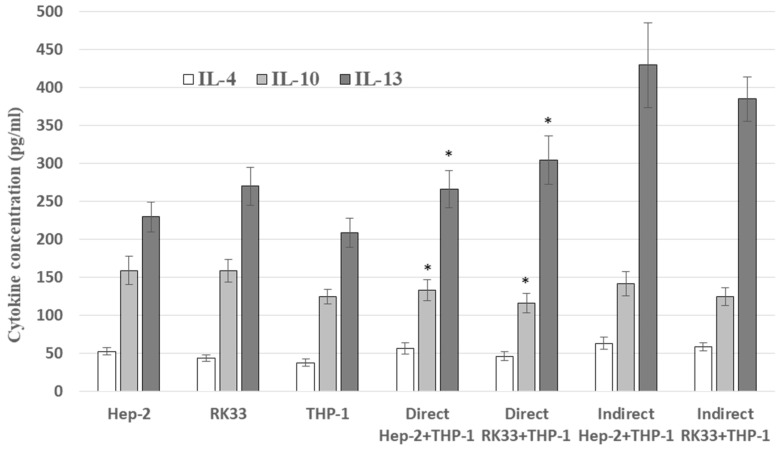
IL-4, IL-10 and IL-13 secretion by the Hep-2, RK33 and THP-1 and tumor/macrophages co-culture (direct and indirect) over 24 h of incubation. ELISA test. Columns and bars show the mean ± standard deviation (n = 3). * *p* ≤ 0.05. Cytokine level in co-culture was compared to the sum of the cytokine released by appropriate cancer cell and monocyte mono-culture.

Direct interactions in the co-culture of Hep-2 or RK33 human cancer cells with THP-1 monocytes were associated with lower IL-13 production than in the corresponding indirect co-cultures in inserts. Although in direct co-cultures a greater amount of IL-13 was demonstrated than in mono-cultures, the total value of the production of this cytokine by both mono-cultures was significantly higher than in co-culture. This indicates the antagonistic activity of the mutual direct relationship between tumor cells and monocytes in relation to the release of IL-13. On the other hand, in indirect co-culture, an additive effect of both cell types in terms of IL-13 production was demonstrated (Figure 2).

There was no difference in IL-10 release in both co-culture systems (direct and indirect). However, a decrease in the production of this cytokine was found in the co-cultures compared to the sum of its production by individual cell types of the respective mono-cultures. It was also lower than the production of IL-10 by the tumor cells alone (Figure 2).

The production of IL-4 did not change when comparing the studied co-cultures with each other and showed no significant changes compared to the corresponding mono-cultures (Figure 2).

In general, the conditions of the co-culture limited the production of the tested cytokines (IL-4, IL-10 and IL-13) as compared to the sum of the amounts of these cytokines obtained in mono-cultures.

### 3.4. Lack of Morphological Changes in Tumor Cells in Tested Co-Cultures

**Figure 3:**
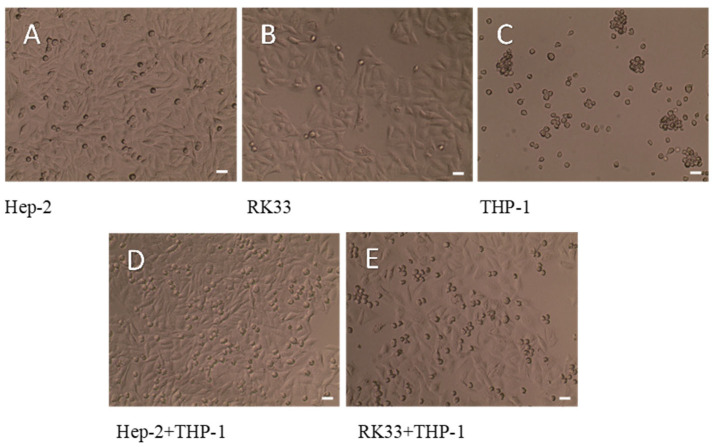
Morphology of human tumor cells Hep-2 (**A**), RK33 (**B**) and human monocyte THP-1 (**C**) in mono-culture. Visualization of THP-1 monocytes with Hep-2 (**D**) and RK33 (**E**) tumor cell co-culture. Preparations were not stained. Images were taken using an Olympus BX51 light microscope. Magnification 100×. Bar = 20 μm.

Cell culture morphology of Hep-2, RK33 and THP-1 as well as their co-cultures without staining is presented in Figure 3.

**Figure 4:**
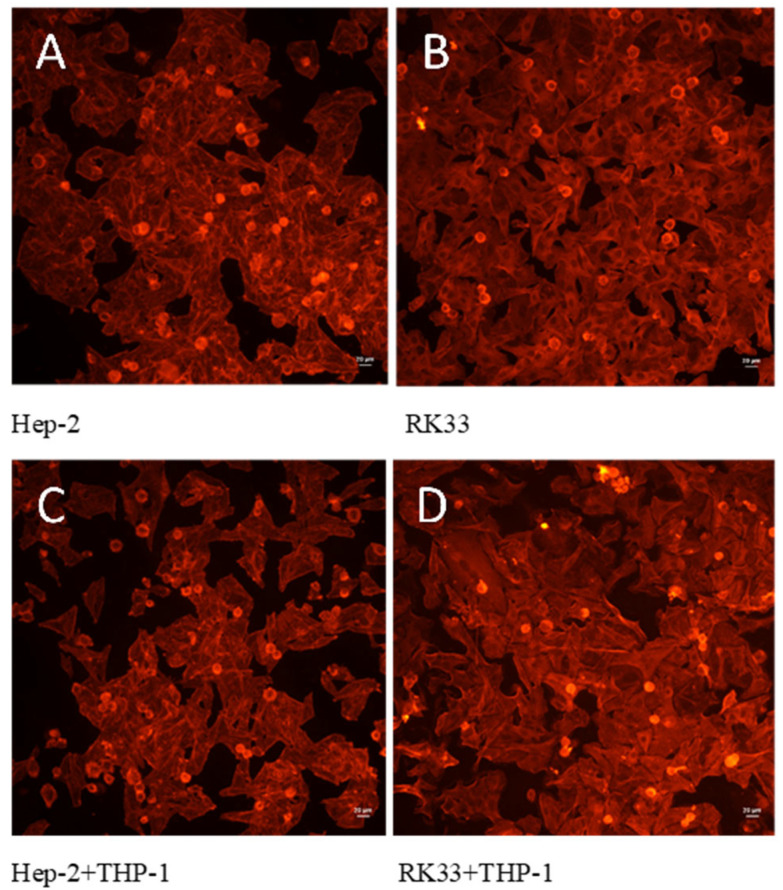
Cytoskeleton F-actin filament organization. Fluorescent staining with Rhodamine–phalloidin dyes. Hep-2 (**A**) and RK33 (**B**) mono-culture. Co-culture of THP-1 monocytes with Hep-2 (**C**) and RK33 (**D**) tumor cells. Images were taken using an Olympus BX51 fluorescent microscope. Magnification 100×. Bar = 20 μm.

No significant changes in the structure of the tumor cell cytoskeleton were observed when comparing the mono-culture of the tumor cells (Hep-2 and RK33) and their co-culture with THP-1 monocytes (Figure 4).

**Figure 5:**
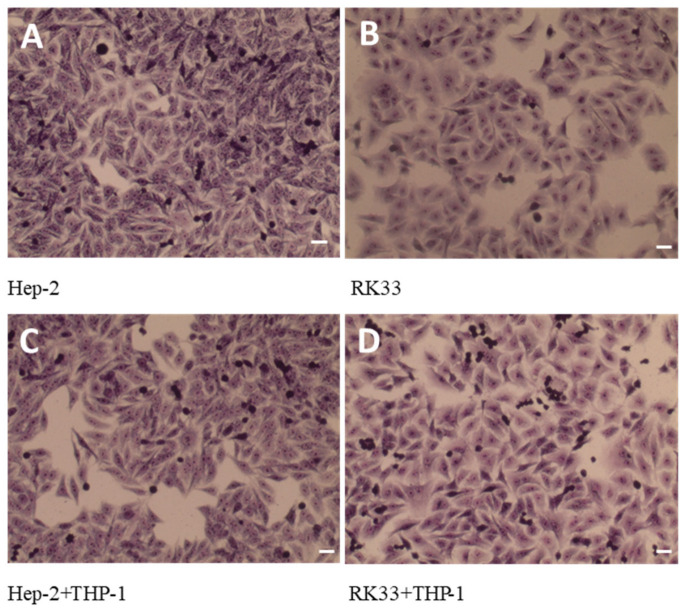
May-Grünwald–Giemsa (MGG) staining. Hep-2 (**A**) and RK33 (**B**) mono-culture. Presentation of the effect of THP-1 monocytes on Hep-2 (**C**) and RK33 (**D**) tumor cell morphology. Images were taken using an Olympus BX51 light microscope. Magnification 100×. Bar = 20 μm.

There were also no significant changes in the morphological changes in the tumor cells growing in the mono-culture as compared to the corresponding co-cultures of these cells with monocytes (MGG staining) (Figure 5).

### 3.5. Fluorescent Analysis of ROS Formation in Tumor/Monocyte Co-Culture

**Figure 6:**
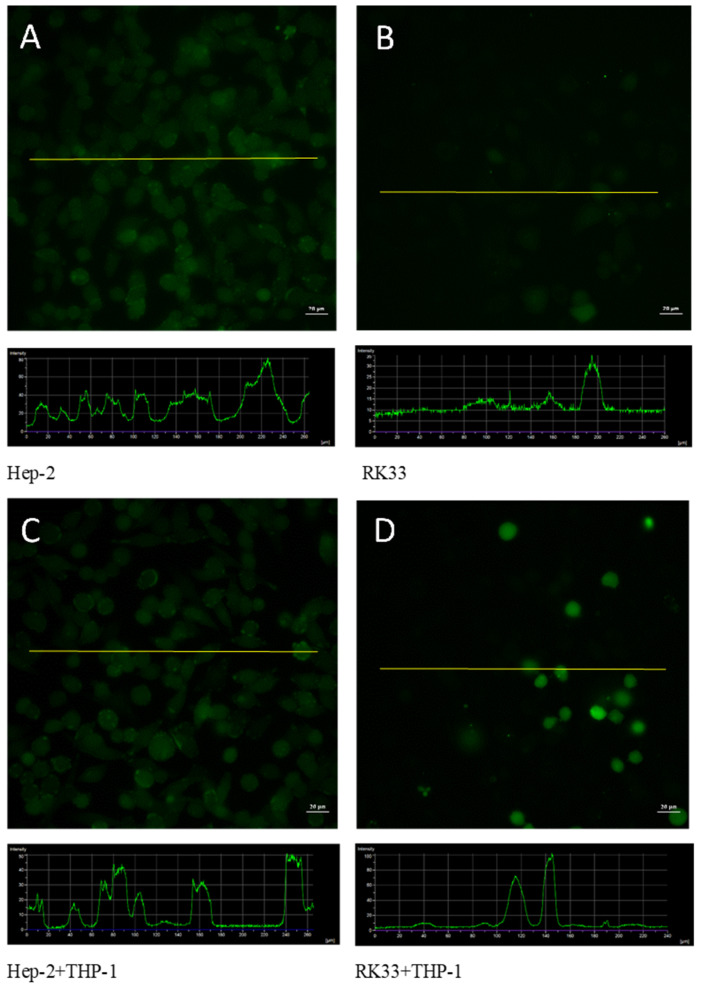
ROS production in co-culture of human tumor cells with human monocytic leukemia cells. Fluorescent staining with 2′,7′-Dichlorodihydrofluorescein diacetate (H_2_DCF/DA). Hep-2 (**A**) and RK33 (**B**) mono-culture. Co-culture of THP-1 monocytes with Hep-2 (**C**) and RK33 (**D**) tumor cells. Images were taken using an Olympus BX51 fluorescent microscope. Magnification 100×. Bar = 20 μm. Representative intensity profiles were shown. Images below the main image preparation show histograms of fluorescence intensity located in specific selected parts of the preparations, which are represented by the line present in the main image. On this basis, the fluorescence intensity could be determined and expressed quantitatively.

The dye intensity was analyzed in the imaging program AnalySIS. The RK33 cells show very low fluorescence resulting from the H_2_DCF/DA dye conversion compared to the Hep-2 cells. The values read on the basis of the fluorescence intensity profile indicated a two-fold difference on average in fluorescence emission. The introduction of monocytes into the Hep-2 cell culture showed no significant changes in the fluorescence of tumor cells alone. However, higher fluorescence was found in the vicinity of cell membranes, which was observed on images and shown in the histograms as more intense emission at the edges of the cell fluorescence peaks. On the other hand, in the case of co-culture of monocytes with RK33 cells, the emission associated with the transformation of the dye mainly by THP-1 cells was found. The fluorescence of tumor cells did not change as compared to the RK33 cell mono-culture. The fluorescence emission of the transformed dye by monocytes was about three times higher than that of tumor cells (Figure 6).

### 3.6. Nitric Oxide Production Decreases in Co-Culture of Tumor Cells with Monocytes

In indirect co-cultures, insignificantly greater amounts of NO_x_ were released as compared to the amount obtained in the direct co-culture system.

**Figure 7:**
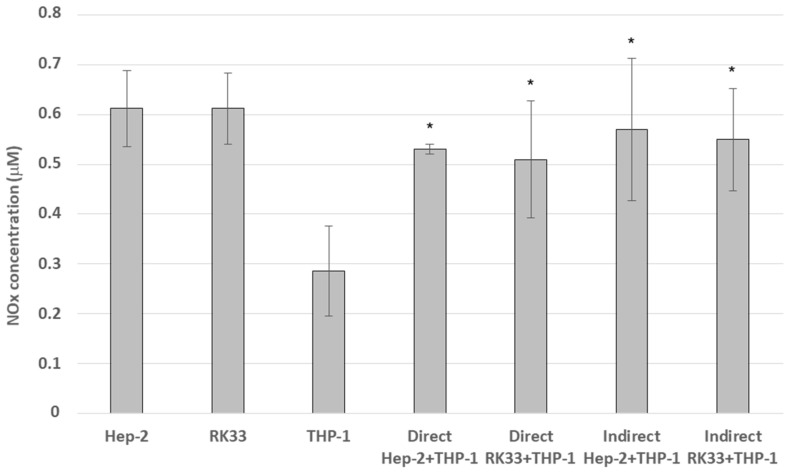
Nitric oxide (NO_x_) secretion in the culture of Hep-2, RK33 and THP-1 mono-cultures and tumor/macrophage co-culture during 24 h of incubation The Griess method. The columns and bars show the mean ± standard deviation (n = 3), * *p* ≤ 0.05. NO_x_ levels produced in co-culture were compared to the sum of NO_x_ released by the appropriate cancer cell and monocyte mono-culture.

In co-cultures, less NO_x_ was released as compared to the sum of the production by both cell types in the mono-culture as well as by the tumor cells alone. The obtained production values indicate that this is an antagonistic effect (Figure 7).

## 4. Discussion

In the presented study, we showed that interactions between tested human cancer cells and macrophages depend on the type of cells and type of cell contact. Modulation of direct interactions through surface receptors (adhesion molecules) and factors produced by both types of cells, as well as indirect interactions mainly through soluble mediators, play an important role in the process of pathological mass development and subsequent metastasis of transformed cells. It was already reported that infiltration of macrophages also causes changes in the expression of selected markers in carcinomas, such as programmed death-ligand 1 (PD-L1). This has a significant impact on the tumor microenvironment and immunosuppression and infiltration by other immune cells such as CD8+ T lymphocytes [14]. Cancer metastasis, as well as subsequent possible recurrences of cancer, may result not only from mutual interactions between cancer cells and their immune microenvironment but also from their mutual abnormal relations [1]. The ways and effects of mutual TAM–tumor cell communications on the overall changes occurring in a tumor mass are not fully understood. However, it is indicated that the biological activity of the local microenvironment, as well as the polarization of macrophages, may affect the shaping of the extracellular matrix (ECM) and thus the epithelial–mesenchymal transition (EMT) and, consequently, the progression of cancer development [9,15,16]. Therefore, direct and paracrine crosstalk between macrophages constituting the largest population of inflammatory cells in tumor stroma and tumor cells, studied in the presented paper, is a contribution designed to show the role of mutual interaction between the tested components of the pathological mass [17]. The role of cancer macrophages is very wide and concerns both the stimulation of angiogenesis, invasion and migration of tumor cells—processes which support cancer stem cells—as well as the acquisition of resistance by the tumor to therapeutic methods such as radiotherapy and chemotherapy [5].

Monocytic cells were used in the conducted research. However, we assumed that under the defined experimental conditions, they are transformed into macrophages. This is just an assumption. The aim of this study was not to determine whether these are M1 or M2 macrophages, but to demonstrate how the interaction between these immune cells and cancer cells under different exposure conditions changes selected parameters related to cancer development. The presented study showed changes in the release of selected cytokines in both tested systems of cancer cells with macrophage interactions. Alternatively activated macrophages are stimulated by cytokines (IL-4, IL-10 and IL-13) produced by helper-2 T cells [18]. In turn, THP-1-derived macrophages stimulate the EMT process and then the invasion and metastasis of cancer cells through the secretion of IL-6. Activation of COX-2/PGE2 factors by this cytokine is also a critical factor in co-culture macrophages/cancer cells, additionally reinforcing pressure on changes in the cellular cytoskeleton and activation of cancer cell mobility [19]. It is indicated that IL-6 present in the co-culture microenvironment stimulates the expression of IL-4 receptors (IL-4R) on cells, sensitizing macrophages to IL-4 activity and thus changing the polarity to M2. Thus, this indicates the important role of both IL-6 and IL-4 in stimulating macrophage polarization in the tumor and finally increasing tumor aggressiveness [5,20]. It was also shown that IL-4 secreted by tumor cells significantly influence tumor infiltrates such as macrophages or CD8 lymphocytes. It enhances cancer-promoting functions by M2 macrophages which are stimulated to produce and activate proteases such as cathepsins [21]. IL-4 upregulates other tumor microenvironmental cytokines including IL-10 which induce immunosuppressive conditions and thus peripheral tolerance of tumor-infiltrating immune cells [22]. IL-10 is secreted both by tumor and immune infiltrates, including lymphocytes and macrophages. We confirmed this in our study. However, in co-culture systems, the level of this cytokine was lower than that observed in tumor cell mono-cultures. According to this, our result can be considered favorable as a high level of IL-10 is correlated with advanced-stage disease and poor prognosis. Nevertheless, this cytokine, through the IL-10/STAT3 or TLR4/IL-10 signaling pathways, promotes the formation of M2 macrophages and activates the expression of the genes responsible for anti-apoptotic, pro-tumor and immunosuppressive activity [7]. Moreover, it is indicated that growth factors and cytokines released by macrophages induce cyclooxygenase-2 (COX-2) in cancer cells. In this context, an increase in IL-10 production would stimulate inflammation within the tumor microenvironment to promote tumor progression [6,10]. In our studies, we demonstrated a relatively high level of released IL-13 in both mono- and co-cultures of tumor cells/macrophages. This cytokine is detected in multiple solid tumors and is associated with cancer progression, among others, due to the fact that it promotes the polarization of M2 macrophages [23]. It has been shown that IL-13 displays biological activity very similar to that of IL-4. This shared biological activity results from sharing common receptor components and Stat6 signal transduction. However, IL-13, much more than IL-4, plays a critical role in the downregulation of tumor immunosurveillance against several types of tumors. It has also been shown that IL-13, by reducing the expression of cadherines, regulates the mobility of cancer cells. Nevertheless, IL-13 does not act as a growth factor but inhibits IL-2-induced tumor cell proliferation and spontaneous apoptosis of cells in tumor masses. In addition, close functional relationships between IL-4 and IL-13 have been demonstrated. The biological activity of IL-13, which is blocked in various ways, is compensated in this respect by IL-4 [24,25]. This may, to some extent, explain the different amounts of both cytokines detected in the presented study. In our work, we did not want to duplicate existing well-established findings nor confirm research available in the literature concerning standard cytokines. We assessed alternative macrophage activation based on the presented cytokines. It turns out that, as a result of the IL-4, IL-13 activity, macrophages acquire an altered M2-type phenotype. It is responsible for changes in the tumor microenvironment and metastasis. It induces inflammation in the tumor niche, among others, due to a local decrease in the concentration of IL-10. We did not want to assess the effect, but the strength of the cause of increased or weakened interactions between the examined cells.

Cytokines released in reciprocal interactions between macrophages and tumor cells significantly affect various mechanisms and processes related to the developing cancer. Among other things, they regulate the inflammatory processes taking place in the tumor microenvironment. However, apart from cytokines, important factors regulating and driving this phenomenon are the local concentration of free oxygen radicals (ROS) and nitric oxide (NO). Hou et al. showed that cytokines released by macrophages can stimulate a ROS/Src/MAPK/AP-1 pathway, which can lead to an increase in COX-2 expression and development of inflammation [26]. In conditions of inflammation, the cytokines released by macrophages stimulate them in an autocrine manner, which not only additionally drives the proliferation of cancer cells or the immunosuppressive status of the tumor but also accelerates cancer metastasis [9,15,27]. In our research, we have shown that the main element of the co-culture that releases free oxygen radicals is the macrophage component. ROS is found to be formed and released mainly in the early stage of monocyte–macrophage differentiation that was simulated in our co-culture model. Generally, generation of ROS by macrophages is not only essential in killing invasive microorganisms but may also be deleterious to surrounding somatic cells. However, ROS are necessary to initiate the differentiation of macrophage cell lines and the occurrence of TAMs. The activity of ERK pathway signal transduction critically regulating monocyte/macrophage differentiation is also under ROS control [28,29]. Excessive ROS microenvironmental concentrations may reduce infiltration tumors via immune cells and accumulation of M2 macrophages. Finally, it may create immunosuppressive local conditions, promoting tumor progression and invasion [30].

Tumor innate immunity mechanisms are also closely connected with nitric oxide (NO) levels. It is already shown that mutual interactions of tumor cells with macrophages strongly influence the progress of a tumor due to, i.a., changes in the generation of nitric oxide (NO). Nevertheless, there are still conflicting results indicating the diverse role of NO within the tumor, which depends on many factors, including the cellular origin of NO, the structure of the tumor, the stage of tumor development or the cytokine composition in the local ECM. Nitric oxide is mainly produced by macrophages, but cancer cells have also been implicated as a source of this molecule. In addition, tumor cells that are close to the hypoxic core are considered to produce less NO/RNS, and it was found that there is a specific gradient of NO/ROS concentrations depending on the location of cells in the tumor mass. In the early stages of tumor development, high levels of reactive nitric species (RNS) initiate cell cycle arrest and induce apoptosis. NO penetration in cancer tissue also results in the accumulation of toxic secondary metabolites that activate the intrinsic apoptotic pathway and may play an important role in CD8+ T cell responses against cancer [31,32]. In turn, in the late stages of tumor development, a decrease in the level of RNS has an anti-apoptotic and beneficial effect for tumor cells. This may result from the activation of S-nitosylation of proteins preventing the degradation of anti-apoptotic proteins, which consequently allows for rapid cell proliferation, proangiogenic effects, immune evasion and thus disease progression [33]. The presence of NO/RNS in the tumor microenvironment regulates the metabolism and functions of macrophages, enabling the development of permanent tumor-promoting inflammation. In addition, NO promotes the maintenance of high levels of pro-inflammatory cytokines in the local environment, exerting a suppressive effect on infiltrating T cell activity [34]. Generally, it can be said that low levels of NO/RNS may exhibit proangiogenic activity that supports immune evasion, whereas high amounts trigger apoptosis.

The architecture of actin cytoskeletons is essential for many features of cancer cells such as maintaining the shape of cells, cell division, migration, adhesion, resistance to chemotherapeutic drugs or anoikis. This is a very dynamic structure involving globular actin (G-actin) turning into fibrillary, filamentous actin (F-actin) and the polymerization and depolymerization of its filaments. In our study, we found no significant changes in the F-actin structure in the co-cultures. However, it is known that G-actin may be a target for selective killing of M2 macrophages and at the same time maintaining high activity of classically activated M1 macrophages showing cytotoxic activity against cancer cells [35].

The cell cycle is a complex process related to cell growth and proliferation. Changes in the course of individual phases of the cycle related to abnormalities in the expression of specific regulatory genes and their products are observed in human cancers. It is indicated that blocking the cell cycle may be an effective strategy to inhibit the uncontrolled growth of cancer cells [36,37]. In the conducted studies, we found changes in the number of cancer cells in individual phases of the cycle depending on the experimental model. Macrophages that polarize towards M2 show endogenous expressions of p53 which regulates not only the cell cycle but also apoptosis, metabolism, DNA repair and tumor immune regulation by controlling the expression of genes involved in these processes. Therefore, it could also affect the division cycle of cancer cells that have had direct or indirect contact with macrophages. Moreover, tumor cells expressing the p53 mutation induce macrophage migration towards M2 [38]. The passage of the cell through the various phases of the cycle is regulated by cyclins and cyclin-dependent kinases (CDKs). Changes in their regulation, especially in the case of cyclin E which binds to and activates cyclin-dependent kinase 2 (CDK2), required for the G1/S-phase transition, could be the cause of the observed changes in the number of cells remaining in G1/S and other phases of the cycle [37].

## 5. Conclusions

In conclusion, the cytokine and reactive forms of oxygen and nitrogen network regulate tumor cell–macrophage interactions. This crosstalk between tumor-associated macrophages and tumor cells may also have a strong impact on pathological mass development modulating functions of different tumor cells. This process, however, differs depending on the kind of cell interaction. Many functional molecules and signaling pathways participating in such interactions can also be accepted as a molecular, therapeutic target to regulate tumor progression. This study showed differences, especially in the level of cytokines (IL-4, IL-13 and IL-10), depending on the model of direct and indirect interactions between laryngeal cancer cells and macrophages. Differences also concern changes in the number of cells in individual phases of the cell cycle. Our interpretation of the cytometric data is based on taking the co-culture as one complete system and is hence a common interpretation of the G1 phase. It may be suggested that the component of direct interactions via surface receptors may regulate the response of cancer cells in co-culture. Indirect interactions, limited only to soluble mediators, are devoid of regulatory mechanisms related to the transmission of signals through surface adhesive particles. Therefore, it may cause the emergence of paracrine relations, which will be a signal for the disclosure of additional stimuli that may increase the instability of cancer cells.

## Figures and Tables

**Table 1:** Changes in LDH release after human tumor cells (Hep-2 or RK33) were co-cultured with THP-1 monocytes relative to the control (Hep-2 or RK33 mono-culture). Direct and indirect (inserts) interactions.

Hep-2 Mono-Culture (% of Cell Death)	RK33 Mono-Culture (% of Cell Death)	THP-1 Mono-Culture (% of Cell Death)	Cell Culture and Type of Interactions	% Change in Co-Cultures Compared to a Cancer Cell Mono-Cultures
5.7% ± 1.6%	----------	9.5% ± 1.4%	Hep-2 + THP-1 (direct)	6.2% ± 3.7% ↓
----------	7.9% ± 0.8%	9.5% ± 1.4%	RK33 + THP-1 (direct)	5.9% ± 3.2% ↑
5.7% ± 1.6%	----------	9.5% ± 1.4%	Hep-2 + THP-1 (indirect)	0.8% ± 3.0% ↓
----------	7.9% ± 0.8%	9.5% ± 1.4%	RK33 + THP-1 (indirect)	12.2% * ± 1.4% ↑

The percent of cell death (% cytotoxicity) was determined using the following equation: % Cytotoxicity = Experimental LDH release (OD570)/Maximum LDH release (OD570). Arrows indicate an increase or decrease in % change in LDH release in co-cultures compared to a cancer cell mono-culture. * *p* ≤ 0.05. Statistical significance in percentage change of released LDH in Co-Cultures Compared to a Cancer Cell Mono-Cultures.

**Table 2:** Statistical analysis of the percentages of human tumor Hep-2 and RK33 and human monocytic THP-1 cells in the sub-G1, G1, S and G2 phases after 24 h mono-cultures or direct and indirect (insert) co-cultures of monocyte and tumor cells.

Cell Cycle	Cell Culture						
	Hep-2	RK33	THP-1	Hep-2 +THP-1	RK33 +THP-1	Hep-2 +THP-1 (Insert)	RK33 +THP-1 (Insert)
Sub-G1	5.99 ± 0.34	7.75 ± 0.71	11.57 ± 0.43	2.59 ± 0.15	6.00 ± 0.59	4.26 ± 0.26	4.15 ± 0.36
G1	67.76 ± 0.54	60.02 ± 0.20	51.29 ± 0.49	68.50 ± 1.01	61.38 ± 1.09	68.88 ± 0.51	62.17 ± 1.29
S	8.40 ± 0.60	18.67 ± 0.24	18.61 ± 0.25	12.95 ± 0.62	20.83 ± 0.93	9.44 ± 0.29	19.67 ± 0.58
G2	18.02 ± 0.39	13.94 ± 0.79	18.88 ± 0.35	16.23 ± 1.12	12.06 ± 0.70	17.50 ± 0.54	14.17 ± 0.97

## Data Availability

Data are contained within this article. Data are also available from the corresponding author on reasonable request.
